# Multi-Channel Fusion Classification Method Based on Time-Series Data

**DOI:** 10.3390/s21134391

**Published:** 2021-06-26

**Authors:** Xue-Bo Jin, Aiqiang Yang, Tingli Su, Jian-Lei Kong, Yuting Bai

**Affiliations:** 1 School of Artificial Intelligent, Beijing Technology and Business University, Beijing 100048, China; jinxuebo@btbu.edu.cn (X.-B.J.); yangaiqiang@st.btbu.edu.cn (A.Y.); kongjianlei@btbu.edu.cn (J.-L.K.); baiyuting@btbu.edu.cn (Y.B.); 2 China Light Industry Key Laboratory of Industrial Internet and Big Data, Beijing Technology and Business University, Beijing 100048, China

**Keywords:** time-series, classification, deep learning, broad learning system, fusion

## Abstract

Time-series data generally exists in many application fields, and the classification of time-series data is one of the important research directions in time-series data mining. In this paper, univariate time-series data are taken as the research object, deep learning and broad learning systems (BLSs) are the basic methods used to explore the classification of multi-modal time-series data features. Long short-term memory (LSTM), gated recurrent unit, and bidirectional LSTM networks are used to learn and test the original time-series data, and a Gramian angular field and recurrence plot are used to encode time-series data to images, and a BLS is employed for image learning and testing. Finally, to obtain the final classification results, Dempster–Shafer evidence theory (D–S evidence theory) is considered to fuse the probability outputs of the two categories. Through the testing of public datasets, the method proposed in this paper obtains competitive results, compensating for the deficiencies of using only time-series data or images for different types of datasets.

## 1. Introduction

The development of sensor technology has increased storage capacity and equipment types and record a significant amount of time-series data. It is very important to perform time-series data analysis in, for instance, accurate classification processing, which is widely used to solve different practical problems, such as mobile object tracking [1], machine fault detection [2], and medical diagnosis [3].

Based on the investigation reported herein, it is found that there are two main time-series classification methods. The first mainly relies on the time series itself, using traditional machine learning or deep learning (DL) for classification. The second kind benefits from the development of image classification networks and encodes time series into images before classification. In this paper, both methods are considered to achieve the use of two modal features. Specifically, long short-term memory (LSTM), the gated recurrent unit (GRU), and bidirectional LSTM (BiLSTM) are selected as the feature extraction method for the original time series due to their ability in automatic feature extraction. Broad learning systems (BLSs) are selected for time-series images, which are simple and satisfy a BLS’s characteristic. In brief, in this paper, a multi-channel fusion classification model is presented to improve the classification effect for different types of series data.

The rest of this article is organized as follows. In Section 2, related work is introduced. In Section 3, the proposed model block diagram and detailed structure are presented. Section 4 presents the experimental data, experimental details, and analysis results. Finally, conclusions are drawn in Section 5.

## 2. Related Work

The processing of classification problems mainly depends on whether the data are similar or not. Time-series classification problems are also analyzed based on this concept. The method of extracting features can be divided into manual and automatic feature extraction for classification.

### 2.1. Methods Based on Manual Feature Extraction

Manual feature extraction is usually used in conjunction with traditional machine learning methods. Measures based on distance are generally adopted, such as Euclidean distance (ED) and dynamic time warping (DTW), and work with k-nearest-neighbor (KNN) classifiers [4]. Huang et al. [5] proposed a KNN algorithm based on class contribution and feature weighting that uses weighted ED to obtain k nearest neighbors. By using class contributions combining the number of k nearest neighbors and their average distance, the final predicted label of samples is obtained. This method achieves a high classification accuracy in tests of public datasets. However, when the data are deformed, such as by scaling, DTW works better than ED. The core idea of DTW is to automatically distort the time series—that is, to perform local scaling on the time axis—so that the shape of the two sequences is as consistent as possible to obtain the maximum possible similarity. Hu et al. [6] selected sample motion data and normalized it to create a template, and then they used the DTW method to compare the processed data with the template to achieve a higher accuracy of activity classification. Furthermore, DTW and ED can work together in the model. Do et al. [7] and Kurt et al. [8] both used the DTW method to align data and then calculated the ED value as one of the metrics for classification. In addition, the hidden Markov model (HMM) [9] and support vector machine (SVM) [10] are also effective machine learning methods. Wang et al. [11] used the Gaussian mixture model to fuse the extracted features and then used the HMM to estimate the output to classify electroencephalogram (EEG) signals. Alickovic et al. [12] proposed a classifier named RotSVM for sleep stage classification, in which the features after noise reduction and discrete wavelet transform are used as input. They built a model that can be effectively used in medical and home care applications.

### 2.2. Methods Based on Automatic Feature Extraction

Although machine learning methods show more superior performance in time-series data classification, many studies have shown that manual feature extraction is not easy with the growth of types and numbers of time series, and traditional machine learning is more suitable for sample learning with lower dimensions. As the superior performance of DL emerges, its application in the time-series analysis is gradually being explored for its ability of automatic feature extraction.

Recurrent neural networks (RNNs) [13,14] are the most commonly used method. Modelling of time-series data by an RNN considers the time correlation of data, which is reflected in the connection of nodes between hidden layers; that is, the input of the hidden layer includes not only the output of the input layer but also the output of the hidden layer at the previous time. In theory, an RNN can process sequence data of any length, but in practice, it is found that it cannot solve the long-term dependence problem. To maintain the memory and dependence on the data, RNN’s variants, LSTM and the GRU were proposed in turn. Dutta [13], compared the simple RNN, LSTM, and GRU with EEG signal data. As the number of layers increases, although it takes longer, the accuracy of the latter two is significantly higher than that of the former. Compared with LSTM, the training time of the GRU is shorter, but the accuracy of the two is comparable. RNN extension methods also include bidirectional LSTM and bidirectional GRU. The bidirectional structure allows the network to consider the context information of time series, and it can show very good results in some tasks, e.g., natural language processing.

In addition to RNN series methods, convolutional neural networks (CNNs) are also used for time-series classification. For example, Kong et al. [15] proposed a fine-grained visual recognition model called MCF-Net to classify different crop species in practical farmland scenes. With multi-stream hybrid architecture utilizing massive fine-granulometric information, MCF-Net obtains preferable representation ability for distinguishing interclass discrepancy and tolerating intra-class variances.

As far as the network architecture is concerned, the characteristics of DL networks are the vertical expansion of the network layer, which imposes a greater demand for computing resources, which, in turn, places higher requirements on hardware. Therefore, in recent years, networks aimed at improving training speed have gradually attracted researcher attention. Among them, BLSs provide an alternative method for DL networks, which also can extract features automatically. Based on a random vector functional link NN (RVFLNN) and incremental learning [16], Chen proposed the BLS [17]. As an efficient incremental learning system without a deep architecture, the wide network can classify images with low background complexity. Based on this finding, Yang et al. applied a BLS to the classification of time-series data and obtained a highly precise classification result [18].

### 2.3. Methods Based on Time-Series Encoding

The aforementioned methods are all from the perspective of data series, which need the memory capacity of the network or the similarity between data to be found through other methods to achieve time-series classification. With the development of DL in image classification, several researchers have discovered ways to encode data from the perspective of images and implement classification. Gramian angular field (GAF) and Markov transition field (MTF) methods proposed by Wang et al. [19] and the recurrence plot (RP) method proposed by Hatami et al. [20] all encode time-series data into images. The advantage is that the time relationship between data points can be directly displayed through images, and then the relationship could use the image classification networks for time-series classification. Inspired by this, Saeed et al. [21] used the GAF method and combined the Inception V3 model to achieve high-precision classification of time series.

## 3. Methods

**Figure 1:**
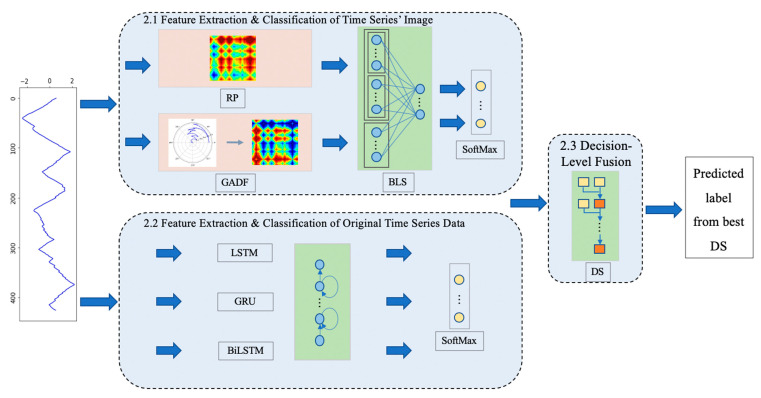
Framework.

The model framework of time-series data combined with multi-modal features presented in this paper mainly includes three parts: time-series data encoding and its feature extraction, original time-series data feature extraction, and decision-level fusion. The specific structural diagram is shown in Figure 1 and described in detail below.

### 3.1. Feature Extraction and Classification of Time-Series Images

In this subsection, the time series is first encoded to images by using RP and GAF, and then the BLS will be used to extract image features. A SoftMax layer is added to obtain the probability result for decision-level fusion.

#### 3.1.1. RP Encoding Method

Inspired by the RP [22], Hatami et al. [20] used two-dimensional phase-space trajectories to visualize time series. RP can analyze the periodicity, chaos, and non-stationarity of time series, reveal the internal structure and give a priori knowledge about similarity, information, and predictability. It is especially suitable for short time-series data. The encoding process is the following.

First, given a time series $X\{x_{1}x_{2},\cdots ,x_{n}\}$ the time-delay embedding method is used to reconstruct the two-dimensional phase space. The state of the phase space with a time delay of 1 is expressed as:

(1) $$s_{1}:\left(x_{1},x_{2}\right),s_{2}:\left(x_{1},x_{2}\right),\cdots ,s_{n-1}:\left(x_{n-1},x_{n}\right)$$

**Figure 2:**
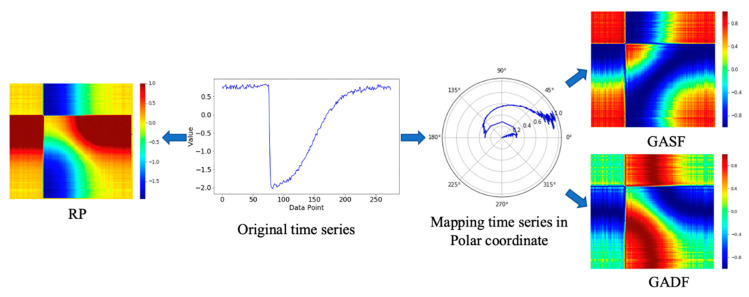
The conversion of a time series using RP and GAF methods.

Then, the RP can be expressed as:

(2) $$R_{ij}=\theta \left(\varepsilon -\left\|s_{i}-s_{j}\right\|\right)$$

where θ(⋅) denotes the Heaviside function, ε is the threshold, and ‖⋅‖ is the norm; an infinite norm is usually used. In the actual encoding, to retain more image details through color transformation, θ(⋅) is not used. In addition, if using an infinite norm for calculation, the image will appear symmetrical, which may make it difficult to distinguish some categories; thus, in this paper, the original difference value using the largest absolute difference value is brought into Equation (2) after comparison using the infinite norm value. Therefore, the RP is expressed as:

(3) $$R_{ij}=\{\begin{array}{c} \varepsilon -\left(x_{i1}-x_{j1}\right),if\left|x_{i1}-x_{j1}\right|\geq \left|x_{i2}-x_{j2}\right|\\ \varepsilon -\left(x_{i2}-x_{j2}\right),if\left|x_{i1}-x_{j1}\right|<\left|x_{i2}-x_{j2}\right| \end{array}$$

where $x_{ik}\;\mathrm{and}\;x_{jk}\left(k=1,2\right)$ represent the kth value of vectors $s_{i}\;\mathrm{and}\;s_{j}$, respectively. The visualization result is shown on the left-hand side of Figure 2.

#### 3.1.2. GAF Encoding Method

The GAF method transfers the normalized series data to a polar coordinate system and then generates the Gramian angular summation field (GASF) or Gramian angular difference field (GADF) matrix by calculating the cosine and sine of the corresponding angle of each pair of elements and then displays the series data in the form of images. The specific conversion process is the following.

Given a time series $X=\left\{x_{1},x_{2},\cdots ,x_{n}\right\}$, since the type of data could be various, it is necessary to normalize the data to intervals [-1, 1] for the same dimension and reduce the amount of calculation,

(4) $${\tilde{x}}_{i}=\frac{\left(x_{i}-\max\left(X\right)\right)+\left(x_{i}-\min\left(X\right)\right)}{\max\left(X\right)-\min\left(X\right)}$$

For each piece of normalized data, the inverse cosine function is used to map to the polar coordinate system and process the time stamp as a radius; the formula is

(5) $$\{\begin{array}{c} \phi_{i}=arc\cos\left({\tilde{x}}_{i}\right),-1\leq {\tilde{x}}_{i}\leq 1,{\tilde{x}}_{i}\in \tilde{X}\\ r_{i}=\frac{t_{i}}{N},t_{i}\in Ν \end{array}$$

where $t_{i}$ is the time stamp, and N is the span of the constant polar coordinate system. In practical applications, its value is equal to the sequence length.

Then, GASF can be defined as:

(6) $$G_{GASF}=\left[\begin{array}{ccc} \cos\left(\phi_{1}+\phi_{1}\right) & \cdots & \cos\left(\phi_{1}+\phi_{n}\right)\\ \cos\left(\phi_{2}+\phi_{1}\right) & \cdots & \cos\left(\phi_{2}+\phi_{n}\right)\\ \vdots & \ddots & \vdots \\ \cos\left(\phi_{n}+\phi_{1}\right) & \cdots & \cos\left(\phi_{n}+\phi_{n}\right) \end{array}\right]$$

and GADF as:

(7) $$G_{GADF}=\left[\begin{array}{ccc} \sin\left(\phi_{1}-\phi_{1}\right) & \cdots & \sin\left(\phi_{1}-\phi_{n}\right)\\ \sin\left(\phi_{2}-\phi_{1}\right) & \cdots & \sin\left(\phi_{2}-\phi_{n}\right)\\ \vdots & \ddots & \vdots \\ \sin\left(\phi_{n}-\phi_{1}\right) & \cdots & \sin\left(\phi_{n}-\phi_{n}\right) \end{array}\right]$$

The above two matrices are used to obtain the images of sequence *X*, as shown on the right-hand side of Figure 2. Through polar coordinate conversion and trigonometric function mapping, the time correlation between different data points is directly displayed by the color of the image.

#### 3.1.3. BLS

**Figure 3:**
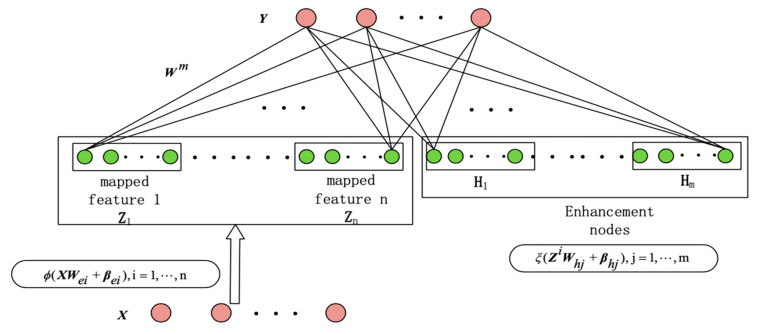
BLS network.

BLS has a variety of structural forms, and the classical structure shown in Figure 3 is used in this paper. It has two kinds of nodes: feature mapping nodes and enhancement nodes. The former performs nonlinear activation on the input data, while the latter, similar to the kernels in convolutional layers, is assumed to be used to fully exploit features in the data and improve the learning ability of the network. The design is the following.

First, the input data are subjected to feature mapping to form a feature node. Second, the feature nodes are enhanced to enhancement nodes by randomly generated weights. The optimal weight selection between the output layer and the feature and enhancement nodes can be obtained by ridge regression and pseudo-inverse algorithms. The specific process is the following.

Assuming that the input data is X with N samples, and each sample has M dimensions. Y is the output matrix that belongs to $R^{N\times C}$. The ith feature mapping groups are represented by

(8) $$Z_{i}=\phi_{i}(XW_{ei}+\beta_{ei})$$

where $\phi_{i}$ is the nonlinear activation function, and $W_{ei}$ is fine-tuned using Equation (9) with iteration steps:

(9) $$\{\begin{array}{c} w_{k+1}={\left(Z^{T}Z+\rho I\right)}^{-1}(Z^{T}x+\rho (o^{k}-u^{k}))\\ o_{k+1}=S_{\frac{\lambda }{\rho }}(w_{k+1}+u^{k})\\ u_{k+1}=u^{k}+(w_{k+1}-o_{k+1}) \end{array}$$

where ρ>0, and S is the soft threshold operator defined as follows,

(10) $$S_{k}(a)=\{\begin{array}{c} \begin{array}{cc} a-k, & a>k \end{array}\\ \begin{array}{cc} 0, & \left|a\right|\leq k \end{array}\\ \begin{array}{cc} a+k, & a<-k \end{array} \end{array}$$

All generated feature nodes are represented by $Z^{n}\equiv \left[Z_{1},\cdots ,Z_{n}\right]$, and then the mth group of the enhancement nodes is represented as:

(11) $$H_{m}\equiv \xi (Z^{n}W_{h_{m}}+\beta_{h_{m}})$$

Therefore, the BLS model can be expressed as:

(12) $$\begin{array}{ll} Y & =\left[Z_{1},\cdots ,Z_{n}|\xi (Z^{n}W_{h_{1}}+\beta_{h_{1}}),\cdots ,\xi (Z^{n}W_{h_{m}}+\beta_{h_{m}})\right]W^{m}\\ & =\left[Z_{1},\cdots ,Z_{n}|H_{1},\cdots ,H_{m}\right]W^{m}\\ & =\left[Z^{n}|H^{m}\right]W^{m} \end{array}$$

where $W^{m}={\left[Z^{n}|H^{m}\right]}^{+}Y$; $W^{m}$ is the connection weight of the broad structure, calculated by the ridge regression algorithm using the following formula to obtain the best value,

(13) $${\left[Z^{n}|H^{m}\right]}^{+}=\underset{\lambda \to 0}{\lim}{\left(\lambda I+\left[Z^{n}|H^{m}\right]{\left[Z^{n}|H^{m}\right]}^{T}\right)}^{-1}{\left[Z^{n}|H^{m}\right]}^{T}$$

### 3.2. Feature Extraction and Classification of Original Time-Series Data

In the preceding subsection, the images encoded from time-series data are used in classification, while the original time series is also considered in order to prevent the information learned from being insufficient.

Time series are limited or infinite data streams that depend on each other between data points, and an RNN is usually used to process such data. In this paper, LSTM, GRU, and BiLSTM are selected as the feature extraction methods of original series data in the way of parallelization, and the SoftMax layer is also added for the later operation of decision-level fusion. The structures of these three methods are introduced in the following subsections.

#### 3.2.1. LSTM

**Figure 4:**
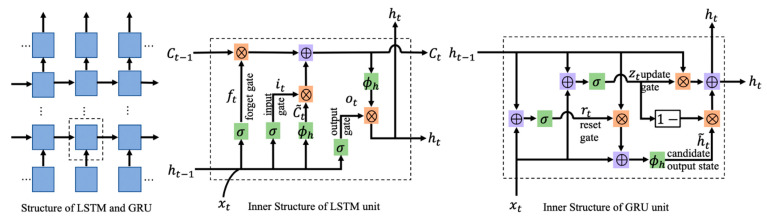
LSTM and GRU networks.

As a special RNN, LSTM is mainly used to solve the problem of gradient disappearance and gradient explosion during long sequence training. In other words, LSTM can perform better in longer sequences than an ordinary RNN. The main reason is that LSTM adds a structure called a gate for selective control of the passage of information. Specifically, it includes three gates, called the forget, input, and output gates. The internal structure of LSTM is shown in Figure 4.

The forget gate is used to determine the retention of the information contained in the previous moment’s state. The input gate selects the new state information that must be added so as to obtain the state of the current moment. The output decides the final unit output at the current time. The Equations of the entire procedure are

(14) $$f_{t}=\sigma \left(W_{fh}h_{t-1}+W_{fx}x_{t}+b_{f}\right)$$

(15) $$i_{t}=\sigma \left(W_{ih}h_{t-1}+W_{ix}x_{t}+b_{i}\right)$$

(16) $${\tilde{C}}_{t}=\tanh\left(W_{Ch}h_{t-1}+W_{Cx}x_{t}+b_{c}\right)$$

(17) $$C_{t}=f_{t}C_{t-1}+i_{t}\times {\tilde{C}}_{t}$$

(18) $$o_{t}=\sigma (W_{oh}h_{t-1}+W_{ox}x_{t}+b_{o}$$

(19) $$h_{t}=o_{t}\times \tanh\left(C_{t}\right)$$

where $f_{t}$ represents the forget gate, $i_{t}$ the input gate, and $o_{t}$ the output gate. σ is the sigmoid function, $h_{t-1}$ the output at the previous moment, *x_t_* the input at the current moment, and *h_t_* the output at current moment.

#### 3.2.2. GRU

Similar to LSTM, GRU is proposed to solve the problems of long-term memory and gradient in back-propagation, but GRU has a simpler structure. It only contains two gates, a reset gate and an update gate, which reduces the amount of calculation it must do. Its internal structure is shown in Figure 4, and the network structure is as same as LSTM.

The reset gate is used to control the degree of ignoring the state information at the previous moment. The smaller the value of the reset gate, the more it is ignored, and the less of the state information is retained. The update gate is used to control the degree of the previous moment’s state being brought into the current state. Different from LSTM, the output of the GRU’s unit contains only $h_{t-1}$, both as the state information of the previous moment to the unit of the next moment and as the input value of the next layer.

(20) $$r_{t}=\sigma \left(W_{rh}h_{t-1}+W_{rx}x_{t}+b_{r}\right)$$

(21) $${\tilde{h}}_{t}=\phi_{h}\left(W_{hh}\left(r_{t}\times h_{t-1}\right)+W_{hx}x_{t}+b_{h}\right)$$

(22) $$z_{t}=\sigma \left(W_{zh}h_{t-1}+W_{zx}x_{t}+b_{z}\right)$$

(23) $$h_{t}=\left(1-z_{t}\right)\times {\tilde{h}}_{t}+z_{t}\times h_{t-1}$$

where $r_{t}$ represents the reset gate, and $z_{t}$ is the update gate. ${\tilde{h}}_{t}$ represents the candidate output value at a current time determined by the reset gate, and $\phi_{h}$ is a hyperbolic tangent function.

Although the structure of GRU is simpler than that of LSTM, the performance of the two is comparable on many tasks. The fewer parameters of GRU make it easier to converge, but when the dataset is large, LSTM may perform better. Therefore, both are considered in this paper.

#### 3.2.3. BiLSTM

**Figure 5:**
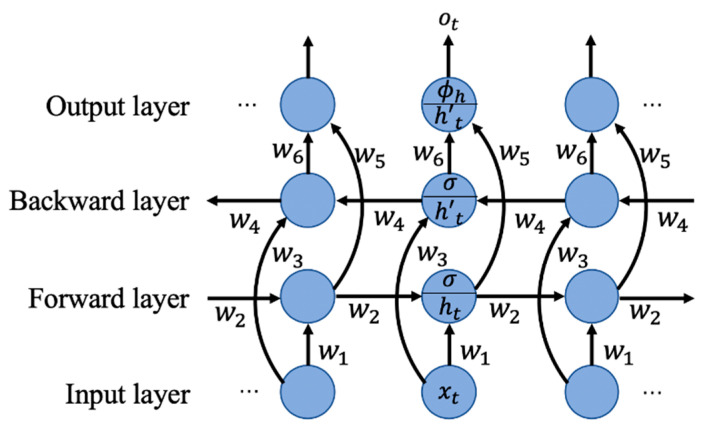
BiLSTM networks.

In addition to the above two RNNs, BiLSTM is also selected as one of the methods. The two-direction structure enables the network to obtain complete past and future context information for each point of the input sequence and can obtain better results in some prediction problems that require context information. The internal structure of BiLSTM is shown in Figure 5.

(24) $$h_{t}=\sigma \left(w_{1}x_{t}+w_{2}h_{t-1}\right)$$

(25) $${h^{\prime }}_{t}=\sigma \left(w_{3}x_{t}+w_{4}h_{t+1}\right)$$

(26) $$o_{t}=\phi_{h}\left(w_{5}h_{t}+w_{6}{h^{\prime }}_{t}\right)$$

where $h_{t}$ is the output of the forward-propagation-layer processing unit at current time t, and ${h^{\prime }}_{t}$ is the output of the back-propagation-layer processing unit.

### 3.3. Decision-Level Fusion

Considering that the abovementioned methods may appear to have different effects on different datasets, to propose a more applicable model, a decision-level fusion strategy was adopted. Specifically, the method of D–S evidence theory is used.

D–S evidence theory is a theory that deals with the uncertainty that was first proposed by Dempster and further developed by G. Shafer. In D–S evidence theory, the required priori data are more intuitive and easier to obtain than in probabilistic reasoning theory. In addition, D–S evidence theory can synthesize the knowledge or data from different experts or data sources. It has the ability to directly express “uncertain” and “unknown. and these pieces of information are represented in the mass function and retained during the evidence synthesis process. These advantages make the D–S evidence theory widely used [23,24]. The theory is defined, and the synthesis process is detailed as follows.

Letting Ω be a recognition frame (or hypothetical space), then the following is defined.

(1) Basic probability allocation (BPA)

The BPA in the recognition framework Ω is a function m, called the mass function, and satisfies,

(27) $$\{\begin{array}{c} m\left(\emptyset \right)=0\\ \sum_{A\subseteq \Omega }m\left(A\right)=1 \end{array}$$

where A is called focal elements that makes m(A)>0.

(2) Belief function

On the recognition framework Ω, the belief function based on m is defined as:

(28) $$Bel\left(A\right)=\sum_{B\subseteq A}m\left(B\right)$$

(3) Plausibility function

On the recognition framework Ω, the plausibility function based on m is defined as:

(29) $$Pl\left(A\right)=\sum_{B\cap A\neq \emptyset }m\left(B\right)$$

(4) Belief interval

In the evidence theory, for a certain hypothesis A in the recognition framework, the BPA is calculated according to the basic probability distribution to calculate the belief function Bel(A) and the plausibility function Pl(A) of the hypothesis to form a belief interval [Bel(A),Pl(A)], which is used to indicate the degree of confirmation of a certain hypothesis.

(5) Dempster’s combinational rule

The combinational rules of mass functions are:

(30) $$\left(m_{1}⊕m_{2}⊕\cdots ⊕m_{n}\right)\left(A\right)=\frac{1}{K}\sum_{A_{1}\cap A_{2}\cap \cdots \cap A_{n}}m_{1}\left(A_{1}\right)\cdot m_{2}\left(A_{2}\right)\cdot \cdots m_{n}\left(A_{n}\right)$$

where K is the normalization constant, calculated by

(31) $$\begin{array}{ll} K & =\sum_{A_{1}\cap \cdots \cap A_{n}\neq \emptyset }m_{1}\left(A_{1}\right)\cdot m_{2}\left(A_{2}\right)\cdot \cdots m_{n}\left(A_{n}\right)\\ & =1-\sum_{A_{1}\cap \cdots \cap A_{n}=\emptyset }m_{1}\left(A_{1}\right)\cdot m_{2}\left(A_{2}\right)\cdot \cdots m_{n}\left(A_{n}\right) \end{array}$$

**Figure 6:**
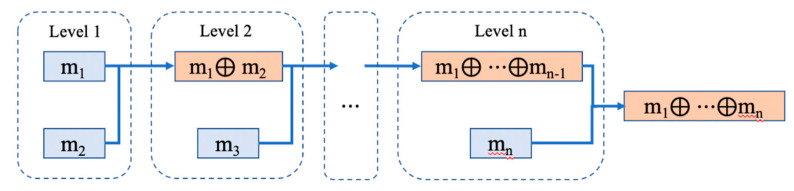
Fusion structure.

In actual fusion, since the predicted label has only one result and there is no overlap, the element of the recognition framework is equal to the actual category of the dataset in this paper, and the probability result of each network for each sample is the mass function of the network. The fusion structure is shown in Figure 6.

## 4. Experiments

The experiments in this paper include two parts. In the first part, the RNN variants are used to classify the original time-series data, and the BLS is used to classify the images and evaluate the accuracy separately. In the second part, decision-level fusion is used to fuse the method results in the first part and evaluate and compare the accuracy.

The data used in this article are from the public time-series dataset UCRArchive_2018 [25], which contains 128 sub-datasets contributed by different researchers. The number of samples and sample length included in each sub-dataset are different, but they are all univariate time-series data, and the training and test sets have been divided. The 128 sub-datasets contain a total of 16 types of data, such as sensor data, edge data of objects in the images, simulation data, and motion data of objects. In this paper, a total of seven sub-datasets in four categories are used to conduct experiments to verify the proposed model. Details of the datasets are given in the following subsections.

### 4.1. BLS and RNN Experiments

#### 4.1.1. BLS Classification Experiment with Encoded Images

In this subsection, both GADF and GASF and RP images are used. During image generation, three image sizes and pixels were fixed. In practice, grayscale images were used for the experiments. The grayscale image reduces the dimension of the input data of the BLS network as well as the amount of calculation relative to the three-channel color image while ensuring recognition accuracy. The BLS network parameters use the same settings as Yang et al. [18]. The number of feature map nodes in each window is 10, there is a total of 10 windows, and the number of feature enhancement nodes is 1500.

As shown in Table 1, using the BLS to classify images of time-series data is effective. However, compared with the GASF method, the overall recognition rate of the GADF method is higher, especially for images that are more difficult to distinguish between classes, such as the image data of SyntheticControl.

**Figure 7:**
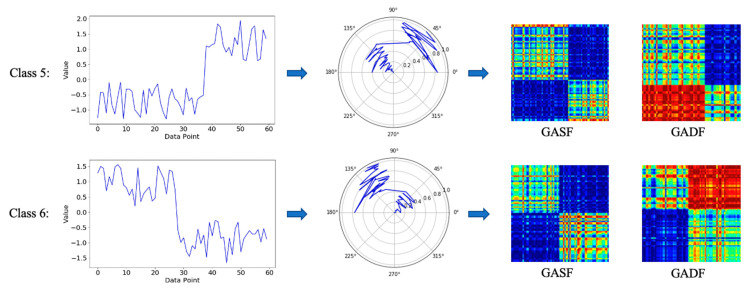
GADF example of stronger image discrimination.

As shown in Figure 7, the samples of the two categories on the left-hand side are completely different from the perspective of timing, and the trends are opposite, but they will be very similar after being reversed. From the polar coordinates in the middle of the figure, the mapped time-series data are also basically symmetrical. The difference between the two data points is between −π and π, and the sum is between 0 and 2π. If the $\cos\left(\phi_{i}+\phi_{j}\right)$ function is used for calculation for two different angles, the order of addition will not affect the calculation result, giving GASF images a high degree of similarity and making it difficult to classify them accurately. On the contrary, if the $\sin\left(\phi_{i}-\phi_{j}\right)$ function is used for calculation, the difference value between two different angles is opposite for two orders so that the calculation result is also the opposite. Therefore, the GADF method can better distinguish such data. The results of the improved RP method in this paper are similar to the GADF results. The main reason is that the two images are similar, and both can distinguish the images very well. However, similar to RNN, the BLS cannot effectively distinguish time series with little difference, such as the depiction of similarly shaped leaves in the OSULeaf dataset. The difference in time-series is quite small, which will lead to overly high image similarity.

A characteristic of the BLS network is that it only needs one epoch of calculation to obtain the result, and once the input and network structure are determined, the result is relatively stable, which is completely different from a DL network. The training result of the latter depends on the setting of network parameters and is prone to fluctuation. In addition, another advantage of BLS is that the training time of one epoch is very short. Even for the OSULeaf dataset, with many samples and long data length, the training time is less than 10 s.

#### 4.1.2. RNN Classification Experiments with Time-Series Data

In this experiment, because the data are not particularly large, to ensure accuracy and as little calculation time as possible, all the three RNN variant network structures in this paper have two hidden layers, a fully connected layer, and a SoftMax layer for classification.

The rules of early stopping have been adopted for the three RNN networks. When training DL networks, the best generalization performance is desired; that is, the data must be well fitted. However, usually, because the hyperparameters are not easy to set, especially the training epoch, the problem of overfitting may occur. Although the network performance improves on the training set and the error rate becomes lower, actually, in some moments, its performance on the testing set has begun to deteriorate. One of the methods that is widely used to solve overfitting problems is to set early stopping rules. The performance of the model is calculated on the validation set during training, and when the performance begins to decline, training is stopped so that the problem of overfitting can be avoided. Since there is no additional validation set in the experiment described in this paper, each generation of the model directly uses all of the testing set to test the performance, and the test accuracy is selected as an indicator of early stopping. To prevent the situation of the training being stopped due to unstable shocks at the beginning, another 75 generations is set when the indicator satisfies the stopping conditions to obtain more stable results.

The proposed network has adopted the Dropout setting, which can prevent overfitting and reduce training time. As is well known, when the number of parameters is increasing, the training speed of the model will be affected obviously. With the Dropout strategy, the resulting training time will be greatly reduced by selectively ignoring some hidden-layer neurons in each epoch. Therefore, Dropout is necessary in our framework for the sake of efficiency.

In addition, the SoftMax activation function is used in the multi-classification problem, and the output is turned into the probability format. As a result, the categorical cross-entropy is chosen as the loss indicator. For the network optimizer, the Adam optimizer is used in the proposed framework. Compared with the Stochastic Gradient Descent (SGD) optimizer, Adam does not need the manually selected initial learning rate, and the optimal value can be automatically adjusted during the training process. Moreover, Adam is easy to implement, computationally efficient, and suitable for scenarios with large-scale data and parameters.

As can be seen from Table 2, the GRU and BiLSTM are superior in terms of accuracy. To further demonstrate the efficiency of the two methods, the averaged time consumption of one epoch for different datasets are compared, and the results are listed in Table 3, where it can be clearly seen that GRU takes obviously less time due to its structural superiority. It needs to be pointed out that the training time might vary with different hardware facilities, experimental environments, etc., in real applications. Although LSTM is not as good as the other two networks in terms of performance, its performance on some datasets is still acceptable, so all three networks are considered for later fusion.

### 4.2. Decision-Level Fusion Experiment and Results Comparison

Considering that different methods exhibit different performances on the same dataset, to ensure that the classification results of images and time series can be reflected in the fusion, a multi-combination fusion method is adopted. At least one result of using time-series data and one of image data is selected for fusion using D–S evidence theory, so there are a total of 13 combinations. The best combination is selected as the final classification result. As shown in Table 4, the best results obtained by fusion are higher than those obtained using a single network in all datasets. Compared with the average accuracy rate, the improvement rate is up to 20.68%. To further verify the performance of the proposed framework, more metrics are introduced to discuss the obtained results. Table 5 shows the results of three evaluation indexes, which are precision rate, recall rate, and F1-score. It can be clearly seen that the proposed model is with the best performance. Among all of the datasets, the averaged precision rate, recall rate, and F1-score are increased by the proposed model with the ratio of 8.5%, 6.82%, and 7.65%, respectively. Thus, the proposed framework is approved to be effective.

## 5. Conclusions

In this paper, BLS is used to classify the images of time-series data, and three recurrent neural networks, i.e., LSTM, GRU, and BiLSTM, were used to classify the time-series data. The BLS and D–S evidence theories are used to combine multiple decision fusion results to select the highest accuracy rate. The results of experiments prove the effectiveness of the proposed framework.

In image classification, the BLS method can quickly and efficiently classify images with lower complexity. Compared with other deep networks, the BLS method can save a significant amount of training time. In terms of overall time usage, the time from encoding time-series data to images to using the BLS for learning is similar, or even less, than using time-series data and RNN variant networks for classification. However, to better improve the applicability of the model to the data, two features are indispensable. In the direct learning and classification of time-series, the series model of RNN is a very good choice due to its memory of the time relationship of the sequence data. LSTM solves the problem of long-term dependence of a traditional RNN through the control of information by forget, input, and output gates, while GRU simplifies the three gates into a reset gate and an update gate. The two performances are similar in most situations. BiLSTM solves the problem of requiring contextual information. In the method of encoding a time series as an image, the GAF and RP methods can intuitively show the time relationship between the sequence data through the image.

Finally, in decision-level fusion, the D–S evidence theory is considered a strategy that can synthesize the results of different decision-making methods; moreover, it does not need to meet the probability additivity requirements. To further improve the classification accuracy, the use of at least one original time-series dataset and one image data results set is guaranteed in this paper, and multi-combination decision-level fusion is carried out to achieve the purpose of fusing the best model.

In future research, the framework proposed in this paper will continue to be improved to solve the problem of fast and efficient classification of multivariate time series.

## Figures and Tables

**Table 1:** The classification accuracy of images from different datasets using the BLS network.

Domain	Dataset	Train	Test	Length	BLS + GASF	BLS + GADF	BLS + RP
Motion	GunPoint	50	150	150	95.33	97.33	93.33
Sensor	Lightning7	70	73	319	61.64	65.75	63.01
	Trace	100	100	275	100	100	96
Image	OSU Leaf	200	242	427	53.31	57.438	58.68
Simulated	SyntheticControl	300	300	60	61.33	97.33	98
	CBF	30	900	128	94.22	95.11	97.22
	UMD	36	144	150	68.75	81.94	97.22

**Table 2:** The classification accuracy of different datasets using RNN series network.

Domain	Dataset	Train	Test	Length	LSTM	GRU	BiLSTM
Motion	GunPoint	50	150	150	97.33	96	97.33
Sensor	Lightning7	70	73	319	58.9	72.6	60.27
	Trace	100	100	275	52	100	71
Image	OSULeaf	200	242	427	57.44	59.92	59.92
Simulated	SyntheticControl	300	300	60	98	99	98
	CBF	30	900	128	98.78	99.11	98
	UMD	36	144	150	79.86	98.61	100

**Table 3:** Averaged time consumption of one epoch for different datasets (Unit: second).

Networks.	GunPoint	Lightning7	Trace	OSULeaf	SyntheticControl	CBF	UMD
GRU	1.13	4.74	4.17	26.56	0.36	1.52	1.05
BiLSTM	1.8	8.2	7.68	47.8	1.53	2.85	1.73

**Table 4:** A comparison of accuracy before and after fusion.

Dataset	LSTM (%)	GRU (%)	BiLSTM (%)	BLS + GADF (%)	BLS + RP (%)	Best D–S (%)	Improvement (%)
GunPoint	97.33	96	97.33	97.33	93.33	98	1.77
Lightning7	58.9	72.6	60.27	65.75	63.01	80.82	20.68
Trace	52	100	71	100	96	100	16.20
OSULeaf	57.44	59.92	59.92	57.438	58.68	66.53	11.80
SyntheticControl	98	99	98	97.33	98	99.67	1.61
CBF	98.78	99.11	98	95.11	97.22	99.67	2.03
UMD	79.86	98.61	100	81.94	97.22	100	8.47

**Table 5:** Evaluation indexes of different models.

Networks		GunPoint	Lightning7	Trace	OSULeaf	SyntheticControl	CBF	UMD
LSTM	Precision	0.9744	0.5908	0.2621	0.5629	0.9807	0.9879	0.8197
	Recall	0.9737	0.5486	0.5	0.5783	0.98	0.9878	0.7986
	F1-score	0.9704	0.5689	0.3811	0.5705	0.9803	0.9879	0.809
GRU	Precision	0.9602	0.746	1	0.6073	0.9901	0.9913	0.9867
	Recall	0.9602	0.7096	1	0.6094	0.99	0.9911	0.9861
	F1-score	0.9602	0.7274	1	0.6083	0.9901	0.9912	0.9864
BiLSTM	Precision	0.9744	0.6239	0.7545	0.5882	0.9803	0.9802	1
	Recall	0.9737	0.4313	0.7267	0.5781	0.98	0.9801	1
	F1-score	0.974	0.51	0.7403	0.5831	0.9802	0.9802	1
BLS + GADF	Precision	0.9735	0.6294	1	0.6018	0.9747	0.9541	0.8167
	Recall	0.9735	0.6048	1	0.5897	0.9733	0.9514	0.8194
	F1-score	0.9735	0.6168	1	0.5957	0.9740	0.9527	0.8178
BLS + RP	Precision	0.9365	0.5456	0.9565	0.6088	0.9809	0.9727	0.9723
	Recall	0.9328	0.5662	0.9643	0.5943	0.9800	0.9724	0.9722
	F1-score	0.9346	0.5557	0.9604	0.6015	0.9804	0.9725	0.9723
Best D–S	Precision	0.9805	0.7950	1	0.6857	0.9967	0.9944	1
	Recall	0.9803	0.6709	1	0.6702	0.9967	0.9945	1
	F1-score	0.9804	0.7277	1	0.6779	0.9967	0.9945	1

## Data Availability

The data supporting reported results can be found in https://www.cs.ucr.edu/~eamonn/time_series_data_2018/, accesseed on 20 September 2019.
